# Diagnostic Performance of an App-Based Symptom Checker in Mental Disorders: Comparative Study in Psychotherapy Outpatients

**DOI:** 10.2196/32832

**Published:** 2022-01-31

**Authors:** Severin Hennemann, Sebastian Kuhn, Michael Witthöft, Stefanie M Jungmann

**Affiliations:** 1 Department of Clinical Psychology, Psychotherapy and Experimental Psychopathology University of Mainz Mainz Germany; 2 Department of Digital Medicine, Medical Faculty OWL Bielefeld University Bielefeld Germany

**Keywords:** mHealth, symptom checker, diagnostics, mental disorders, psychotherapy, mobile phone

## Abstract

**Background:**

Digital technologies have become a common starting point for health-related information-seeking. Web- or app-based symptom checkers aim to provide rapid and accurate condition suggestions and triage advice but have not yet been investigated for mental disorders in routine health care settings.

**Objective:**

This study aims to test the diagnostic performance of a widely available symptom checker in the context of formal diagnosis of mental disorders when compared with therapists’ diagnoses based on structured clinical interviews.

**Methods:**

Adult patients from an outpatient psychotherapy clinic used the app-based symptom checker *Ada–check your health* (ADA; Ada Health GmbH) at intake. Accuracy was assessed as the agreement of the first and 1 of the first 5 condition suggestions of ADA with at least one of the interview-based therapist diagnoses. In addition, sensitivity, specificity, and interrater reliabilities (Gwet first-order agreement coefficient [AC1]) were calculated for the 3 most prevalent disorder categories. Self-reported usability (assessed using the System Usability Scale) and acceptance of ADA (assessed using an adapted feedback questionnaire) were evaluated.

**Results:**

A total of 49 patients (30/49, 61% women; mean age 33.41, SD 12.79 years) were included in this study. Across all patients, the interview-based diagnoses matched ADA’s first condition suggestion in 51% (25/49; 95% CI 37.5-64.4) of cases and 1 of the first 5 condition suggestions in 69% (34/49; 95% CI 55.4-80.6) of cases. Within the main disorder categories, the accuracy of ADA’s first condition suggestion was 0.82 for somatoform and associated disorders, 0.65 for affective disorders, and 0.53 for anxiety disorders. Interrater reliabilities ranged from low (AC1=0.15 for anxiety disorders) to good (AC1=0.76 for somatoform and associated disorders). The usability of ADA was rated as high in the System Usability Scale (mean 81.51, SD 11.82, score range 0-100). Approximately 71% (35/49) of participants would have preferred a face-to-face over an app-based diagnostic.

**Conclusions:**

Overall, our findings suggest that a widely available symptom checker used in the formal diagnosis of mental disorders could provide clinicians with a list of condition suggestions with moderate-to-good accuracy. However, diagnostic performance was heterogeneous between disorder categories and included low interrater reliability. Although symptom checkers have some potential to complement the diagnostic process as a screening tool, the diagnostic performance should be tested in larger samples and in comparison with further diagnostic instruments.

## Introduction

### Background

Digital technologies represent an increasingly important source of health information. Approximately 6 out of 10 European adults use the internet to seek health information [1]. Meanwhile, internet search engines can be considered a common starting point for self-diagnosis, which can have a significant effect on health care decisions and outcomes. The popularity of web-based health information seeking arises from the ease of access and immediacy of a plethora of health resources in various formats (eg, encyclopedias, blogs, social media, video channels, health apps, and telemedicine). Diagnosis websites could promote early diagnosis and help-seeking, which in turn may lead to earlier treatment and thus prevent chronic courses.

Mental health topics are among the most popular search queries [1], and it is estimated that approximately one-third of all health apps worldwide target mental health issues [2]. The use of these digital health resources may have various structural and individual reasons. For example, individuals who feel stigmatized or ashamed by mental health issues (eg, obsessive-compulsive symptoms and sexual dysfunctions) could benefit from anonymity and low-threshold information [3,4]. Interpersonal communication problems, often associated with severe mental disorders, can become barriers to traditional help-seeking and may also turn patients toward digital resources. In addition, there is considerable uncertainty in the population regarding the significance and pathological threshold of mental health issues [5]. Access to adequate treatment and diagnosis is often complicated and delayed (eg, concerns about psychological treatment, long waits, and restricted availability of psychotherapy in rural areas) [6,7].

Although digital health resources can ideally increase access to health care and empower patients to engage in health behavior [8], the information provided is mostly unregulated and can also contain confusing or unsubstantiated facts and recommendations [9]. This could promote incorrect self-diagnosis and problematic health decisions [10]. A study by Grohol et al [11] on the quality of web-based mental health information revealed that 67.5% of 440 investigated websites contained information of good or better quality. However, the quality of information varied between disorders, and readability was rated as difficult. For anxiety disorders, another study found only a poor-to-moderate quality of internet-based information [12]. In addition, many websites also showed a lack of or inadequate information regarding a rough classification of symptoms and possible health care professionals or services to contact [13]. Similarly, studies from the mobile health app database project rated the overall information quality of apps for various mental disorders (eg, depression and posttraumatic stress disorder) as poor to mediocre and found that only a fraction had been evaluated scientifically [14,15].

Selecting, interpreting, and using web-based health information requires sufficient eHealth literacy [16]; however, this can be unevenly distributed across age, socioeconomic, or educational groups, which has been termed “digital divide” [17]. Thus, a substantial proportion of internet users may experience difficulties in web-based health information seeking, and individuals with chronic health problems who may have a particular need for information and support are seemingly less likely to obtain helpful information [18]. Users typically rate the internet “higher as a source to use than a source to trust” [19], particularly when compared with personal medical information (eg, from health professionals). In addition, digital health information may lead to increased illness anxiety [20], which in turn increases unnecessary health care use and costs [21,22]. In this regard, health professionals are also facing new challenges (eg, biased expectations and less trust in medical advice) with internet-informed patients [23].

### Symptom Checkers for Condition Suggestion and Triage Advice

An emerging alternative to internet search engines is the so-called symptom checkers, which aim to provide rapid and differentiated condition suggestions and assistance with the urgency of care advice. Symptom checkers typically use dynamically structured interviews or multiple-choice questions and, as a result, provide one or more condition suggestions, usually ranked by their likelihood (eg, 7 *out of 10 persons with these symptoms have been diagnosed with this condition*). The mostly algorithm-based programs typically operate with chatbots to simulate a dialogue-like human interaction [24]. Symptom checkers can also be used as a diagnostic support system for health professionals [25]. General diagnostic and triage advice of specific symptom checkers has been studied for a broad range of general and specialized health problems [26], for example, ophthalmologic [27] or viral diseases [28,29].

Research indicates that, although symptom checkers seem to be easy to use and well-accepted by most users [30,31], the diagnostic performance varies significantly between different symptom checkers and has been interpreted as low to moderate at best [32,33]. Semigran et al [34] investigated the diagnostic accuracy of 23 symptom checkers using 45 standardized case vignettes of various health conditions that would require emergent care (eg, appendicitis and heart attack) or nonemergent care (eg, back pain), or where self-care would be appropriate (eg, bronchitis). Across symptom checkers, the correct diagnosis was listed first in only 34% of cases, with considerable performance variation between symptom checkers (5%-50%). A similar average performance rate was found for a broader set of 200 clinical vignettes in a recent study that compared the condition suggestion accuracy of 8 popular symptom checkers (*Ada–check your health* [ADA], Babylon, Buoy, K Health, Mediktor, Symptomate, WebMD, and Your.MD) with diagnoses obtained from general practitioners for various health conditions, including some mental health issues [35]. The investigated symptom checkers showed a highly variable diagnostic coverage, from 99% (ADA) to 51.5% (Buoy). Significant differences in condition suggestion accuracy were observed between symptom checkers, with accuracy for the first listed condition suggestion ranging from 19% (Symptomate) to 48.5% (ADA) with an average of 26.1%. The symptom checkers listed the correct diagnosis in the top 5 condition suggestions in 40.8% of cases, whereas the best accuracy was reported for ADA (77.5%). However, these findings should be interpreted cautiously as most authors were employees of Ada Health GmbH. Most recently, a study by Ceney et al [33] yielded comparable average performance rates (51%, range 22.2%-84%) for the top 5 condition suggestions of 12 symptom checkers based on case vignettes.

In contrast to patients’ rather positive perspectives on the usability and utility of symptom checkers, health professionals seem to be more skeptical [25], and symptom checkers have had an inferior performance compared with professional diagnoses in previous studies [32]. According to a review by Semigran et al [36], 84.3% of physicians’ top 3 diagnoses matched those of clinical vignettes compared with 51.2% of symptom checkers (*P*<.001). Generally, diagnostic performance seems to converge when the number of diagnostic suggestions taken into account is increased. For example, ADA reached a similar diagnostic accuracy to general practitioners (77.5% vs 82.8%) when considering the range of the top 5 diagnostic suggestions in the study by Gilbert et al [34]. In another study, the Babylon Diagnostic and Triage System reached comparable diagnostic sensitivity (80%) with physicians (83.9%) [37]. However, various methodological concerns regarding this study have been raised, such as sensitivity to outliers [38]. In a Spanish study, 622 patients at a tertiary care university hospital emergency department responded to the questions of the symptom checker Mediktor. The physicians’ diagnoses matched 1 of the first 3 diagnoses of Mediktor in 75.4% of cases and the first diagnosis in 42.9% of cases. Again, as this study was conducted by committed future company members of the investigated symptom checker at the time of publication, findings should be interpreted cautiously.

Although previous studies mostly cover a range of physical conditions (which most symptom checkers were primarily designed to detect), the usability and diagnostic performance in mental disorders have not been investigated sufficiently. A recent pilot study by Jungmann et al [39] investigated the performance and dependency on expert knowledge of the symptom checker ADA in diagnosing mental disorders in adults and adolescents. Psychotherapists, psychology students, and laypersons entered symptoms from case vignettes into the app. For mental disorders in adulthood, the diagnostic agreement between the textbook diagnoses and the main condition suggestion by the app was moderate (68%) but increased to 85% when ADA’s differential diagnoses were taken into account. Diagnostic agreement with case vignettes was higher for psychotherapists (79%) than for psychology students (58%) or laypersons (63%), demonstrating the beneficial effect of expert knowledge.

### Objectives

Notably, previous studies on symptom checkers have relied primarily on standardized case vignettes, which are less likely to represent real-world cases with clinical comorbidity and, as such, may overestimate the diagnostic accuracy of symptom checkers. Furthermore, the diagnostic quality at the consumer level (ie, patients rather than health professionals) has been insufficiently studied but is of paramount interest for a robust evaluation of the accuracy of symptom checkers in clinical settings. Therefore, this study aims to evaluate the diagnostic performance of a widely available symptom checker when used by patients compared with diagnoses by psychotherapists using structured clinical interviews.

## Methods

### Design

This study was designed as an observational, comparative, prospective study in adult outpatients conducted at the psychotherapy outpatient clinic of the University of Mainz (Germany). In the outpatient clinic, >1400 patients are treated per year on average by approximately 160 therapists. The study was conducted in compliance with ethical principles and approved by the ethics committee of the Department of Psychology at the University of Mainz (2019-JGUpsychEK-009, June 28, 2019).

### Participants and Recruitment

Participants were recruited consecutively between August 2019 and December 2020 in the outpatient psychotherapy clinic of the University of Mainz. Inclusion criteria were age ≥18 years and sufficient knowledge of the German language. We excluded patients with acute suicidality (assessed by a score of ≥2 on item 9 of the Beck Depression Inventory-II [40]), patients with any self-indicated acute mental or physical state (eg, psychosis or brain injury) that would prevent safe and meaningful use of the app, and patients who did not receive a diagnosis of a mental disorder by therapists in the diagnostic interview. Diagnoses were obtained from 42 experienced therapists. At the time of the study, the therapists were in advanced cognitive behavioral therapy training (≥1.5 years of clinical practice) and had completed a 2-day training course on the use of structural clinical interviews.

### Procedure

After having indicated interest in participating in the trial, participants were screened for inclusion with a web-based questionnaire and received detailed information on the study. Eligible participants provided written informed consent to participate. Consequently, the participants were asked to fill out a demographic questionnaire. During their waiting time before their initial appointment at the outpatient clinic, the participants were then invited to answer the questions of the symptom checker on a 10-inch tablet. The patients were instructed to focus on the current most disturbing mental health symptoms. Patients and therapists were not informed about the condition suggestions by the app until the completion of the diagnostic interviews so that the subsequent diagnostic process would not be influenced. For this purpose, the patients were instructed to stop using the symptom checker before the condition suggestions were displayed. The therapists were informed about the study and routinely performed the German version of the Structured Clinical Interview for the Diagnostic and Statistical Manual of Mental Disorders, Fourth Edition (SCID) [41], during the initial therapy sessions, which can be considered a *gold standard* of the diagnosis of mental disorders in research along with individually selected self-report instruments. The therapists were asked to report their diagnoses back to the study team and were then unblinded and informed about the symptom checker’s condition suggestions, which they discussed with the patient to allow for professional clarification of ambiguous or contradictory results. For compensation, the patients could participate in a raffle of gift certificates (5 × €20 [US $22.91]), and the therapists were reimbursed with €5 (US $5.73) per case.

### Instruments

#### App-Based Symptom Checker

The symptom checker ADA (Ada Health GmbH) is a Conformité Européenne–certified medical device assisting in the screening of medical conditions. For this purpose, ADA is available at the consumer level as a self-assessment app [42], whereas a prototype diagnostic decision support system for health professionals has been developed as well [43]. This particular app was selected for various reasons: (1) the diagnostic coverage is wide [35], including mental disorders, and ADA has shown acceptable diagnostic performance in this diagnostic spectrum recently [39]; (2) it is free of charge and widely available (>10 million users and 7 languages) for Android- and iOS-running devices [42]; (3) it provides probabilities for a list of differential condition suggestions; (4) in comparison with other symptom checkers, it has performed more accurately in formal diagnosis [34,35]; and (5) it has proven to be well-accepted and easy to use in a large sample of primary care patients [30].

ADA is based on a dynamic medical database, which is updated through research findings and app entries [44]. Using artificial intelligence, a chatbot asks questions in various formats (eg, open questions with text-based answers and discrete items) about current symptoms. Standard questions include age, gender, smoker status, presence of pregnancy, high blood pressure, and diabetes. As a result, ≥1 condition suggestion is determined to best match the pattern of symptoms entered. The user is presented with a probability of possible diagnoses (eg, 6 out of 10 people with these symptoms have a social anxiety disorder), including a list of other less probable condition suggestions (see [45] for an example process). Finally, the app offers information on the urgency of medical help-seeking (eg, urgent care needed). In this study, version 3.1.2 of ADA was used.

#### Usability

The usability of the symptom checker was assessed using the 10-item, unidimensional System Usability Scale (SUS) [46], a widely used, reliable scale [47]. The items (eg, *I find the app easy to use*) are rated on a 5-point Likert scale (0=*strongly disagree* to 4=*strongly agree*). Reliability was acceptable in this study (McDonald *ω*=0.72). Furthermore, an adapted version of a 15-item questionnaire, which was previously used to investigate the usability of a computerized standardized clinical interview [48], was implemented. For the purpose of this study, 12 items were selected, which could be answered on a 4-point Likert scale (1=*strongly disagree* to 4=*strongly agree*). Reliability was acceptable in this study (*ω*=0.74). Both questionnaires were completed as paper and pencil versions after completion of the symptom checker.

#### Additional Measures

Further items covered demographic characteristics (age, gender, mother tongue, relationship status, and educational level), clinical characteristics (symptom duration, history of mental disorder diagnoses, and psychotherapeutic treatments), previous experience with ADA (yes or no), and frequency of web-based health information seeking (*Do you use the Internet to inform yourself about symptoms of your mental health problems?* with answers from 0=*never* to 3=*always*). The time required to complete the diagnostic process in the app and the number of questions asked until completion were assessed.

### Statistical Analyses

All text diagnoses were recoded into International Classification of Diseases, 10th Revision (ICD-10), codes (as a universal medical coding system) by a trained clinical psychologist not otherwise involved in the study and cross-checked by another clinical psychologist at the Masters level (97.1% agreement). Disagreements between the raters were resolved by including a third licensed therapist (first author).

The condition suggestions were compared with the therapists’ diagnoses at the level of 4-digit codes in the ICD-10 (eg, F40.1, social phobia). Following the procedure by Jungmann et al [39], if the fourth digit represented a more detailed specification (eg, F32.2, major depressive disorder, single episode, severe without psychotic features), the 3-digit code match was counted for the following disorders: depressive disorder, bipolar affective disorder, obsessive-compulsive disorder, conduct disorder, or schizophrenia. For the diagnosis of agoraphobia with panic disorder (F40.01), both the condition suggestions *agoraphobia* and *panic disorder* were counted as accurate. The condition suggestion *Burnout* was coded as a depressive disorder. As condition suggestions to our knowledge did not include recurrent depressive episodes (F33.X), these diagnoses were treated as equal to the nonrecurrent category (F32.X). Furthermore, the terms *abuse* and *addiction* were judged to agree as the app did not distinguish between abuse and addiction to our knowledge. Functional somatic syndromes (eg, fibromyalgia and irritable bowel syndrome) were associated with somatoform disorders (F45) [49]. Analyses of the agreement were assessed for both the total sample and disorder categories (first 2 ICD-10 digits, eg, affective disorders and anxiety disorders). We noted whether the symptom checker’s first condition suggestion or any of the first 5 of the symptom checker’s condition suggestions (including *less probable condition suggestions* if not >5 in total) matched any of the interview-based diagnoses to assess diagnostic accuracy. For example, we counted a correct diagnosis listed first if a patient was diagnosed with agoraphobia with panic disorder (F40.01) and specific phobia (F40.2) by therapists using the SCID and ADA’s top 1 condition suggestion was *panic disorder (7 out of 10)*. Accuracy was calculated as the percentage of agreement along with the 95% CI for binomial distributions with the Agresti-Coull method [50]. For the 3 most prevalent disorder categories in our sample (according to the interview-based diagnoses), we calculated accuracy based on contingency tables as the sum of true positives and true negatives divided by the total number of cases [51], as well as sensitivity and specificity. In addition, the Gwet first-order agreement coefficient (AC1) [52] was calculated to assess interrater reliability. The AC1 is less prone to overcorrection for chance agreement and less sensitive to low base rates compared with other coefficients such as the Cohen *κ* [52,53]. Values <0.20 indicate poor strength of agreement, 0.21-0.40 indicate fair strength of agreement, 0.41-0.60 indicate moderate strength of agreement, 0.61-0.80 indicate good strength of agreement, and >0.81 indicate very good strength of agreement [54].

Scores on the SUS were calculated by subtracting 1 from the raw scores of odd-numbered items and, for the even-numbered items, by subtracting the raw score from 5 and multiplying the sum of these adjusted scores by 2.5 [55] (score range 0-100). According to Bangor et al [56], scores >70 are considered acceptable, and ≥85.5 is considered excellent. Scores for the feedback questionnaire were analyzed at the item level. Missing values in both usability questionnaires were infrequent (maximum of 2/49, 4% per variable) and were replaced with multiple imputations using a Markov chain Monte Carlo algorithm with 5 imputations per missing one. The imputed data sets were merged to obtain 1 data set. Associations between completion time of ADA and patient characteristics were explored using bivariate correlations. The AC1 was calculated using AgreeStat version 2011.3 (Advanced Analytics). All other analyses were performed using SPSS (version 27; IBM Corp) and *α*=.05 as a level of significance.

## Results

### Study Flow

Over the 1.5-year recruitment period, 159 persons were screened for inclusion, of which 104 (65.4%) did not meet the inclusion criteria or did not provide informed consent. Of the remaining 55 study participants, 6 (11%) had no interview-based diagnoses available because of early discontinuation of treatment; thus, complete data were available for 49 (89%) study participants. Table 1 shows the demographic and clinical characteristics of the participants. On average, the participants were 33.41 (SD 12.79) years old, and 61% (30/49) were women. Approximately 22% (11/49) of participants reported using the internet *often* or *always* for health information search. The mean symptom duration was 8.25 (SD 8.22) years, and 39% (19/45) of participants with available data reported past diagnoses of mental disorders.

**Table 1:** Demographic and clinical characteristics of the participants (N=49).

Variable		Values
Age (years), mean (SD, range)		33.41 (12.79, 18-66)
**Gender, n (%)**		
	Female	30 (61)
	Male	19 (39)
**Level of education, n (%)**		
	Primary level	3 (6)
	Intermediate level	28 (57)
	Higher level	17 (35)
	Other degrees	1 (2)
**Family status, n (%)**		
	Single	33 (67)
	Married or permanent partnership	15 (31)
	Divorced, living apart, or widowed	1 (2)
**Mother tongue, n (%)**		
	German	46 (94)
	Language other than German	3 (6)
Duration of symptoms (years), mean (SD)		8.25 (8.22)
**History of mental disorders,^a^ n (%)**		
	Affective disorders	10 (22)
	Anxiety disorders	9 (20)
	Other disorders	6 (13)
	No history of mental disorders	30 (67)
Past psychotherapy (yes), n (%)		25 (51)
**Web-based health information seeking, n (%)**		
	Never	8 (16)
	Rarely	30 (61)
	Often	10 (20)
	Always	1 (2)

^a^n=45. Multiple answers possible.

### Diagnostic Agreement

On average, 2.06 (SD 0.99) diagnoses by the therapist and 3.44 (SD 1.06) condition suggestions by ADA were recorded per patient. Approximately 67% (33/49) of patients received >1 diagnosis. The most prevalent diagnostic categories in our sample (101 therapist diagnoses for 49 cases) were affective disorders (F30-F39; 34/101, 33.7%), anxiety disorders (F40-F41; 27/101, 26.7%), and somatoform and associated disorders (including F45; 9/101, 8.9%). Multimedia Appendix 1 contains a detailed list of interview-based diagnoses and ADA’s condition suggestions.

In 51% (25/49; 95% CI 37.5-64.4) of cases, ADA’s first condition suggestion was in accordance with any of the therapists’ diagnoses, and it was in the top 5 condition suggestions in 69% (34/49; 95% CI 55.4-80.6) of cases. When considering the frequency of comorbid diagnoses, on average, ADA was able to detect <1 (mean 0.80, SD 0.64) of the mean 2.06 (SD 0.99) therapist diagnoses per patient.

Table 2 displays the performance statistics of the symptom checker’s condition suggestions for the 3 most common disorder categories. The highest accuracy was observed in somatoform and associated disorders (0.76 to 0.82), and the lowest was observed in anxiety disorders (0.45 to 0.53). Sensitivity was highest for affective disorders (0.65 to 0.71) and lowest for somatoform and associated disorders (0.22 to 0.29). Interrater reliabilities (AC1) ranged from low strengths of agreement for anxiety disorders (−0.09 to 0.15) to moderate-to-good strengths of agreement for somatoform and associated disorders (0.65 to 0.76) according to proposed benchmarking thresholds [54].

**Table 2:** Performance statistics of *Ada–check your health* (ADA) for disorder categories.

Performance statistics	Correct condition suggestion by ADA						
	Listed first				Listed in top 5		
	Affective disorders	Anxiety disorders	Somatoform + associated disorders	Affective disorders		Anxiety disorders	Somatoform + associated disorders
Accuracy (95% CI)	0.65 (0.51 to 0.77)	0.53 (0.39 to 0.66)	0.82 (0.68 to 0.90)	0.63 (0.49 to 0.75)		0.45 (0.32 to 0.59)	0.76 (0.62 to 0.86)
Sensitivity	0.65	0.21	0.22	0.71		0.43	0.33
Specificity	0.67	0.84	0.95	0.50		0.46	0.85
AC1^a^ (95% CI)	0.32 (0.46 to 0.60)	0.15 (−0.16 to 0.47)	0.76 (0.59 to 0.93)	0.31 (0.26 to 0.60)		−0.09 (−0.39 to 0.20)	0.65 (0.44 to 0.86)

^a^AC1: Gwet first-order agreement coefficient.

Separately, we examined the diagnostic accuracy of ADA for the level of severity of mild or moderate and severe depression (without cases with partially or fully remitted recurrent depression) as indicated by the therapists’ diagnoses. ADA listed the correct (severity) condition suggestion first in 44% (10/23; 95% CI 25.6-63.2) of cases and in the top 5 condition suggestions in 61% (14/23; 95% CI 40.7-77.9) of cases.

### Usability

None of the participants indicated having used ADA before. The average completion time of ADA was 7.90 (SD 3.39) minutes, and an average of 31.90 (SD 8.11) questions were asked. Completion time was significantly positively associated with age (*r*=0.40; *P*=.004) and illness duration (*r*=0.41; *P*=.004) but not with frequency of web-based health information seeking (*r*=−0.10; *P*=.497) or level of education (*r*=0.03; *P*=.85) and did not differ with gender (*t*_47_=0.53; *P*=.60). On average, the participants rated the usability on the SUS as high (mean 81.51, SD 11.82), with significantly lower values in male compared with female participants (mean difference −8.61, SE 3.28; *t*_47_=−2.63; *P*=.009). Usability was significantly negatively associated with age (*r*=−0.41; *P*=.003) but not with illness duration (*P*=.86), frequency of web-based health information seeking (*P*=.53), or level of education (*P*=.57).

Table 3 shows the item statistics for the feedback questionnaire [48]. Approximately 88% (43/49) of participants were satisfied with how they answered ADA’s questions, 61% (30/49) found that ADA’s questions were clear to them, and 71% (35/49) would have preferred a face-to-face interview.

**Table 3:** Item descriptions for the feedback questionnaire (adapted from Hoyer et al [48]).

Item number^a^	Item	Agreement,^b^ n (%)
1	Sometimes I could not follow the app’s instructions.	11 (22)
2	I enjoyed answering the questions.	34 (69)
5	Throughout the questioning, my concentration was good.	46 (94)
6	The questions were clear to me.	30 (61)
7	Now and then I wanted to quit the questioning.	1 (2)
8	The questioning was a pleasant experience for me.	37 (76)
9	During the questioning, my endurance was steady.	47 (96)
10	I’m satisfied with how I answered the questions.	43 (88)
12	I did not understand how the questions were related to my problems.	2 (4)
13	Anything related to apps makes me feel uncomfortable or anxious.	3 (6)
14	I would have preferred a normal face-to-face interview from patient to therapist.	35 (71)
15	I think it was good that the questioning was done in such an exact and detailed manner.	40 (82)

^a^Number of original items. Items 3, 4, and 11 were excluded from this study.

^b^Aggregated frequency of answers (4) *completely agree* and (3) *agree*.

## Discussion

### Principal Findings

To our knowledge, this comparative study is the first to independently investigate the diagnostic accuracy of a popular symptom checker (ADA) as a screening tool for mental disorders compared with validated formal diagnoses in real-world patients. Our results show that, in approximately half of all investigated cases (25/49, 51%), ADA’s first listed condition suggestion was correctly aligned with any of the interview-based expert diagnoses. This transdiagnostic accuracy was higher than the average rates of symptom checkers from previous comparative studies (26%-36%) that used case vignettes of various health conditions [34,36,57]. Furthermore, the accuracy observed in our study is close to the performance rate of ADA (48.5%) across a broad spectrum of medical conditions in the study by Gilbert et al [34] but lower than in another recent comparative study (72%) [35]. When compared with a study by Barriga et al [58], who investigated the accuracy of another symptom checker (Mediktor) in real patients in an emergency care unit, the accuracy for the first listed condition suggestions was in a comparable range (51% vs 42.9%). In two-thirds (34/49, 67%) of cases, 1 in 5 condition suggestions aligned with any of the interview-based diagnoses, which is somewhat below the range of performance rates of ADA in previous studies using case vignettes (77%-84%) [34,35] or patients seeking emergency care (91.3%) [58]. However, our findings can only be compared with the accuracy from previous studies to a limited extent. These studies included only 1 potentially correct diagnosis per case as opposed to multiple diagnoses per case in our study.

The transdiagnostic accuracy of ADA could be considered lower when compared with sensitivities of self-report screenings for mental disorders that range between 0.72 and 0.90 according to previous studies [59-62]. However, the different measures of agreement must be considered here. Interestingly, the transdiagnostic performance of ADA when used by patients is comparable with that of studies in which medical experts used ADA to enter information based on case vignettes [34]. This is in contrast to previous findings by Jungmann et al [39], who demonstrated lower performance rates of ADA in laypeople compared with health professionals with regard to correctly identifying mental disorders from case vignettes of adults and adolescents. However, our study was designed differently as we did not use standardized vignettes, and therapist diagnoses were not checked by independent raters. An interesting future study design would be to directly compare the expert and consumer-level use of symptom checkers and explore differences in diagnostic performance. However, we provide preliminary evidence that no expert knowledge or user experience may be needed to yield performance rates comparable with those of health professionals using symptom checkers. As our participants were all novices in the use of ADA, we could not test the potential beneficial effect of familiarity on diagnostic accuracy. Future studies could, for example, include a test run where participants enter information from a standardized vignette to familiarize themselves with the symptom checker.

Within the most prevalent subcategories of mental disorders in our sample, we observed considerable differences in performance statistics. For somatoform and associated disorders, accuracy, specificity, and interrater reliabilities were highest and could be considered acceptable. This may resemble the accuracy of ADA, particularly in detecting somatic medical conditions, which has been the focus of previous studies [34,35]. Beyond this, the unifying classification of functional somatic syndromes (eg, irritable bowel syndrome and fibromyalgia) as somatoform disorders is subject to ongoing controversial debate [49,63]. However, the base rate (<10%) was lowest across disorder categories, which in turn may have inflated specificity and interrater reliability. For affective and anxiety disorders, performance was lower than one would expect given that these disorder categories have a high prevalence in the general as well as clinical populations [64,65] and when compared with higher sensitivities of self-report screenings, particularly those observed for anxiety disorders [66-68]. However, with regard to the small sample size, and as the diagnostic coding scheme [39] could be considered relatively liberal for some disorders, replication in a larger sample and with more fine-grained diagnostic coding seems warranted to obtain a more robust estimation of diagnostic performance.

Furthermore, the participants rated the usability of ADA as high, which is in line with data from a previous study in primary care patients [30]. However, self-selection of study participation could have positively biased usability ratings. Concerning acceptability, almost three-fourths of our participants (35/49, 71%) preferred face-to-face diagnostics by a health professional over the symptom checker, which is comparable with preference ratings from the German general population [18]. This could be critical regarding the *reshaping* of diagnostic practice as acceptance represents a crucial premise for the implementation of health resources [69]. As symptom checkers are more likely to complement rather than substitute diagnostic processes, it would be interesting to also investigate patients’ and health professionals’ views on the combination of traditional and digital diagnostic procedures, for example, whether symptom checkers would be preferred as a first or second opinion in differential diagnoses or as assistance in clinical decision-making. In this regard, we did not confront the patients or therapists directly with the condition suggestions to not influence the diagnostic process. However, for clinical implementation, it would be interesting to study how symptom checkers used early in the patient journey preempt the diagnostic process and medical decisions. Further studies could also investigate the trust of users in the diagnostic and triage suggestions of symptom checkers compared with other sources of health information (eg, the internet and health professionals).

### Strengths and Limitations

Concerning the interpretation of our results, several limitations must be considered. Generally, the therapists’ diagnoses were based on additional information beyond the diagnostic interview (eg, anamnesis, medical records, and questionnaires) that was not available to the symptom checker, which represents a much more extensive process in terms of time and content, whereas, in using the symptom checker, the patients could decide what and how many different symptom complexes they entered. Although this ensured a user-oriented research focus, findings on diagnostic accuracy must thus be interpreted against the informational disbalance between the 2 rating sources. In this regard, it should also be noted that we compared ADA’s *differential* condition suggestions for 1 symptomatology with final diagnoses by therapists (and not vice versa with their differential diagnoses). Thus, it seems reasonable to remind clinicians that expect symptom checkers to be a universal screening tool that these are designed to provide condition suggestions for 1 symptomatology at a time and, given their current intended purpose, are not suited to replace a broad diagnostic screening (eg, via validated questionnaires or interviews). Furthermore, as digital resources may change over time, particularly when considering learning algorithms, current accuracy rates may do so as well. As previous studies have shown considerable differences between symptom checkers’ diagnostic accuracy [33,35], future studies could compare various symptom checkers for the formal diagnosis of mental disorders. On this matter, evidence indicates that the use of algorithms over other methods, the inclusion of demographic information [57], or more rigorous questioning [35] could explain the differences between symptom checkers’ diagnostic performances.

In addition, as this study had a pilot character and pandemic restrictions further impeded recruitment, we included a rather small sample when compared with previous studies with patients [58]. Large-scale, multicenter studies are warranted for more robust estimates of diagnostic performance, including a more fine-grained analysis of unprocessed diagnoses. The diagnostic spectrum of our participants was somewhat limited (Multimedia Appendix 1), with substance abuse disorders, eating disorders, or posttraumatic stress disorders being underrepresented. However, the most common mental disorders were frequent in our sample and resembled prevalence rates in medical settings [70]. In contrast to previous comparative studies [34], we did not include >1 diagnostic rater or assess the correctness of interview-based diagnoses. Previous studies have demonstrated a large variation in interrater reliabilities of diagnoses based on SCIDs that can range from substantial to even low agreement [71-73], which may challenge the validity of this as a *gold standard* in diagnosis [74].

Although the therapists who participated in this study were in advanced clinical training, including diagnostic training and regular supervision, and thus were experienced in performing diagnostic procedures, we did not assess the level of (diagnostic) experience or check the therapists’ or symptom checker’s diagnoses independently. In addition, newer versions of diagnostic systems (eg, the Diagnostic and Statistical Manual of Mental Disorders, Fifth Edition, and the ICD-11) and corresponding clinical interviews should be considered as comparators in further research. Generally, one could also criticize the exclusive categorical diagnostic approach of this study, which has been challenged recently by a strictly empirical and dimensional understanding and taxonomy of psychopathology such as the Hierarchical Taxonomy of Psychopathology [75], and dimensional self-report instruments would be a logical comparator for future studies.

However, our study constitutes a robust test of the diagnostic accuracy of ADA in comparison with formal clinical diagnostics, which is pivotal for clinical implementation. We considered some major limitations of previous studies [32] given that we collected real-world patient data, which comes closer to the current intended laypeople-oriented application of symptom checkers. In contrast to standardized vignettes, which have been the default method in previous studies, our data were thus not limited to single-diagnosis cases and included consistent comorbidities. In addition, we were able to recruit a diverse sample, which covered various age groups as well as intensities of health-related internet use. Eventually, we performed an independent scientific evaluation of a commercially available product, which seems important given the plethora of health apps that have not been scientifically reviewed [14,15].

### Clinical Implications

Our findings offer various clinical implications. At the public health level, symptom checkers have some potential to reduce underdiagnosis and undertreatment of mental disorders [76] and may ideally contribute to reducing chronicity and treatment delay as they represent a low-threshold, multilingual diagnostic instrument. For their possible role in formal diagnosis, the level of diagnostic and triage accuracy is the most important indicator. However, for individuals with mental health problems, the exact differentiation (eg, the severity of major depression and type of anxiety disorder) could be less important than informing on the broader diagnostic category and providing triage advice. Here, evidence shows that, although most symptom checkers seem to provide safe triage advice [33], they are somewhat more risk-averse [57] than health professionals, which could increase health care use and costs. Then again, when compared with entering symptoms into a web-based search engine, symptom checkers are likely to be a superior tool for diagnostic assistance. However, both sources can have a similar risk of adverse emotional or behavioral consequences according to a recent study by Jungmann et al [20]. For example, similar to a search engine, a symptom checker can increase health anxiety and negative affect after searching for causes of symptoms (eg, shortness of breath). In addition, symptom checkers could make the diagnostic process less intuitive and controllable, and vulnerable patient groups, less educated people, or older people are probably less likely to take advantage of this resource at the public health level, thus increasing the “digital divide” [77,78].

As argued by Semigran et al [33], if symptom checkers are regarded as a potential replacement for professional diagnostics (ie, beyond their current intended purpose), they are likely an inferior alternative. Although the average diagnostic performance of symptom checkers can be considered generally low when compared with diagnostic standards (eg, expert diagnoses and validated diagnostic instruments), some symptom checkers show more promising performance rates, including the symptom checker studied here [34,35]. Nevertheless, the progressive dissemination of smart screening instruments may contribute to shared decision-making and promote patients’ understanding of and engagement in health decisions. As such, digital health resources have already become an important factor in the therapist-patient relationship [79] as more patients use digital resources for diagnostic and treatment purposes.

Although symptom checkers or even automated (eg, avatar-based) diagnostic systems [80] may reduce clinician time, they still rely on the active engagement of users. The advancement of passive mobile sensing through smartphones or wearables (eg, mobility pattern, facial expression, and speech analysis [81,82]) may allow for in situ, fine-grained digital phenotyping even without this active user input. Although this may reduce the diagnostic effort, at the same time, the perceived control over the diagnostic process could be limited. Thus, both active and passive diagnostic approaches will have to demonstrate their quality and acceptability in routine care.

Besides their potential as a waiting room screening tool, the most typical use case would be to study users in their home environment. This would also allow for a better understanding of adequate medical help-seeking, which seems to be positively associated with the triage advice of symptom checkers [83].

Finally, future research should address the effect of symptom checkers on other meaningful outcomes, such as stigmatization, attitudes toward psychotherapy, health-related self-efficacy, or the association with treatment success, which would advance the understanding of the clinical impact of these tools on mental health care.

### Conclusions

Overall, our findings indicate that the diagnostic performance of a widely available symptom checker in detecting mental disorders in real patients is close to the range of performances from previous case vignette studies that covered a broad spectrum of medical conditions. From a formal diagnostic standpoint, ADA could provide clinicians with a list of condition suggestions with moderate-to-good accuracy, whereas diagnostic performances were inconsistent between disorder categories and also included low interrater reliabilities. The symptom checker was rated as user-friendly overall but was less preferred than face-to-face diagnostics. The value of symptom checkers for diagnostic screening needs to be tested on larger samples and in comparison with further diagnostic resources such as established self-report screenings.

## Appendix

Multimedia Appendix 1 Interview-based expert diagnoses and condition suggestions by the symptom checker app (*Ada–check your health*).

## Abbreviations

AC1: Gwet first-order agreement coefficient
ADA: *Ada–check your health*
ICD: International Classification of Diseases
SCID: Structured Clinical Interview for the Diagnostic and Statistical Manual of Mental Disorders, Fourth Edition
SUS: System Usability Scale
